# Case report: splicing effect of a novel heterozygous variant of the NUS1 gene in a child with epilepsy

**DOI:** 10.3389/fgene.2023.1224949

**Published:** 2023-07-04

**Authors:** Yan Hu, Mingwei Huang, Jialun Wen, Jian Gao, Weiwei Long, Yansheng Shen, Qi Zeng, Yan Chen, Tian Zhang, Jianxiang Liao, Qiuli Liu, Nannan Li, Sufang Lin

**Affiliations:** ^1^Department of Neurology, Shenzhen Children’s Hospital, Shenzhen, China; ^2^ Aegicare (Shenzhen) Technology Co., Ltd., Shenzhen, China

**Keywords:** NUS1, trio-WES, aberrant splicing, epilepsy, case report

## Abstract

*NUS1* is responsible for encoding of the Nogo-B receptor (NgBR), which is a subunit of cis-prenyltransferase. Over 25 variants in *NUS1* have been reported, and these variants have been found to be associated with various phenotypes, such as congenital disorders of glycosylation (CDG) and developmental and epileptic encephalopathy (DEE). We report on the case of a patient who presented with language and motor retardation, epilepsy, and electroencephalogram abnormalities. Upon conducting whole-exome sequencing, we discovered a novel pathogenic variant (chr6:118024873, NM_138459.5: c.791 + 6T>G) in *NUS1*, which was shown to cause Exon 4 to be skipped, resulting in a loss of 56 amino acids. Our findings strongly suggest that this novel variant of *NUS1* is responsible for the development of neurological disorders, including epilepsy. It is believed that the truncation of Nogo-B receptor results in the loss of cis-prenyltransferase activity, which may be the underlying cause of the disease.

## Introduction

The *NUS1* (nuclear undecaprenyl pyrophosphate synthase 1) gene encodes for the Nogo-B receptor (NgBR), a highly conserved protein with multiple functions (Grabinska et al., 2017; Den et al., 2019; Riboldi et al., 2022). NgBR acts as a subunit of cis-prenyltransferase (cis-PTase) and plays a critical role in promoting isoprenyltransferase activity by interacting with dehydrodolichyl diphosphate synthase (DHDDS) (Harrison et al., 2011; Park et al., 2014; Giladi et al., 2022). Previously, a genetic mutation in *NUS1* has been identified in a family with a congenital glycosylation disorder. The mutation was homozygous and resulted in a missense variant. Patients with this disorder exhibited various neurological symptoms, including psychomotor retardation and refractory epilepsy (Park et al., 2014; Jaeken and Peanne, 2017). The discovery of the genetic basis for *NUS1* has opened up avenues for studying its role in dolichol synthesis and protein glycosylation. So far, 28 pathogenic or likely pathogenic *NUS1* gene variants have been reported, with 17 of these having traceable clinical descriptions (Szafranski et al., 2015; Hamdan et al., 2017; Guo et al., 2018; Araki et al., 2020; Wirth et al., 2020; Courage et al., 2021; Fraiman et al., 2021; Gunzler and DeBrosse, 2021; Haginoya et al., 2021; Yu et al., 2021; Zhang et al., 2021; Monfrini et al., 2022; Ji et al., 2023). These studies have found that pathogenic variations in *NUS1* can cause a range of diseases, many of which are associated with epilepsy, such as myoclonus, tremor, ataxia, and dystonia, among others. Here, we report the case of a patient with a novel pathogenic *NUS1* splice variant, characterized by seizures with myoclonus and axial tonics, fine motor and intellectual impairments, and abnormal electroencephalography.

## Materials and methods

### Patient and clinical assessments

The patient received medical care at Shenzhen Children’s Hospital, where various clinical diagnostic examinations, including MRI and EEG, were conducted. Medications prescribed for the patient included levetiracetam, sodium valproate, zonisamide, and nitrazepam. Additionally, the patient was assessed using the Wechsler Intelligence Scale, received ketogenic diet therapy, and underwent adjunctive therapy with a skin patch. Furthermore, trio whole-exome sequencing tests were performed.

### Trio whole-exome sequencing

Genomic DNA was extracted from peripheral blood samples. Trio whole-exome sequencing (Trio-WES) was performed using a SureSelect Human All Exon kit V6 (Agilent Technologies, Santa Clara, CA) on the Illumina Novaseq6000 at an average depth of 100× at Berry Genomics, Beijing, China.

### RNA-Seq analysis

RNA extraction was carried out using TRIzol reagent, ensuring the retrieval of total RNA. To enrich the mRNA, a subsequent step was performed, followed by purification. The purified mRNA was then fragmented, and reverse transcription was conducted to synthesize complementary DNA (cDNA). The generated cDNA underwent end repair and 3′-adenylation processes. Adapter sequences were ligated to the cDNA molecules, facilitating their amplification and purification through PCR. The PCR products were then converted into single-stranded DNA, which were further transformed into closed-loop structures. Subsequently, a sequencing library was prepared, and finally, sequencing was performed.

### Reverse transcriptase PCR analysis

Total RNA was extracted from peripheral blood using the Blood (Liquid Sample) Total RNA Rapid Extraction Kit (RP4001, Bioteke); this was followed by genome digestion and cDNA reverse transcription using the HifairTM 1st Strand cDNA Synthesis SuperMix (11123ES70, YEASEN) kit. The system was heated to 95°C for 5 min, followed by PCR amplification using PrimerSTAR MAX DNA Polymerase (R045A, TaKaRa) at 95°C for 30 s, 57°C for 30 s, and 72°C for 90 s (35 cycles), and then 72°C for 5 min. Finally, Sanger sequencing was performed to verify the results.

### Minigene assay

To analyze how the identified variants affected splicing, minigene assay was carried out; the specific steps were as follows. First, minigene trapping and digestion site introduction were performed. Second, the recombinant vectors were transiently transfected into HeLa and 293T cell lines; the transfection step was performed according to the liposome instructions (Rapid Plasmid Mini Kit, 1005250, SIMGEN) and the samples were collected after 48 h (DNA Gel Extraction Kit, 2001250, SIMGEN). Next, total RNA was extracted from cell samples using an RNA extraction kit (Trizol RNAiso PLUS, 9109, TaKaRa, Kusatsu, Japan). After determination of the concentration, cDNA was synthesized by reverse transcription with equal amounts of RNA. Finally, PCR amplification was performed using the flanking primers (pcDNA3.1-F and pcDNA3.1-R) on the minigene vector, and the resulting amplified gene transcript bands were detected via agarose gel electrophoresis. Each band was then recovered separately for Sanger sequencing.

## Results

### Case report

The patient was a 12-year-old girl and the first child to non-consanguineous, healthy parents, born normally. Her birth weight was 3150 g. She began to walk independently at 14 months of age. No obvious abnormalities were observed before the age of two. At 2 years old, rapid shaking and tremor of hands when holding objects were observed. The symptoms were aggravated during mood fluctuations and were dramatically improved upon administration of nitrazepam.

When the patient was 6 years old, she developed a frequent rapid hand tremor; therefore, she underwent monitoring via brain magnetic resonance imaging (MRI) and electroencephalography (EEG) at a local hospital. Brain MRI was normal, but EEG revealed interictal generalized spike-and-wave patterns and myoclonic seizures. The patient was treated with levetiracetam and sodium valproate, which resulted in improvement in symptoms. In addition, moderate myopia was noticed at the time.

**FIGURE 1:**
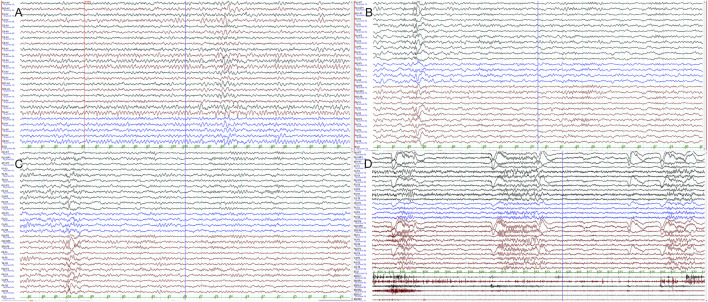
Clinical presentation: electroencephalograms. EEG monitoring of the patient at 10 years of age revealed slow posterior predominant activities **(A)**, interictal occipitoparietotemporal spikes and waves **(B,C)**, and frequent blinking in clusters accompanied by low-amplitude, generalized fast activities **(D)**.

At 10 years old, the patient presented with frequent blinking, accompanied with slightly worsened cognition and school performance, with a Wechsler intelligence score of 77. She could walk and speak normally. Although she still had slight tremor during fine hand movements, she could write in Chinese that was easily recognizable. EEG monitoring revealed tonic seizures: in terms of semiology, these presented as frequent blinking in clusters along with low-amplitude, generalized fast activities in ictal EEG (Figure 1). Repeated brain MRI was normal. The patient was switched to a ketogenic diet, which was followed by improvement in cognition and a slight reduction in blinking. Trio whole-exome sequencing and mitochondrial gene sequencing were carried out. She also received perampanel add-on therapy, resulting in a 60% reduction in blinking frequency and slight improvement in limb tremor. Zonisamide was given to aid in further control of tonic seizures, which produced a limited reduction in seizure frequency. At the age of 12, her height was 152 cm (−0.2 SD) and her weight was 32 kg (−1.8 SD). She continued to exhibit blinking and hand tremor and was taking perampanel, zonisamide, and nitrazepam.

### Identification of a heterozygous splicing site variant in NUS1

**FIGURE 2:**
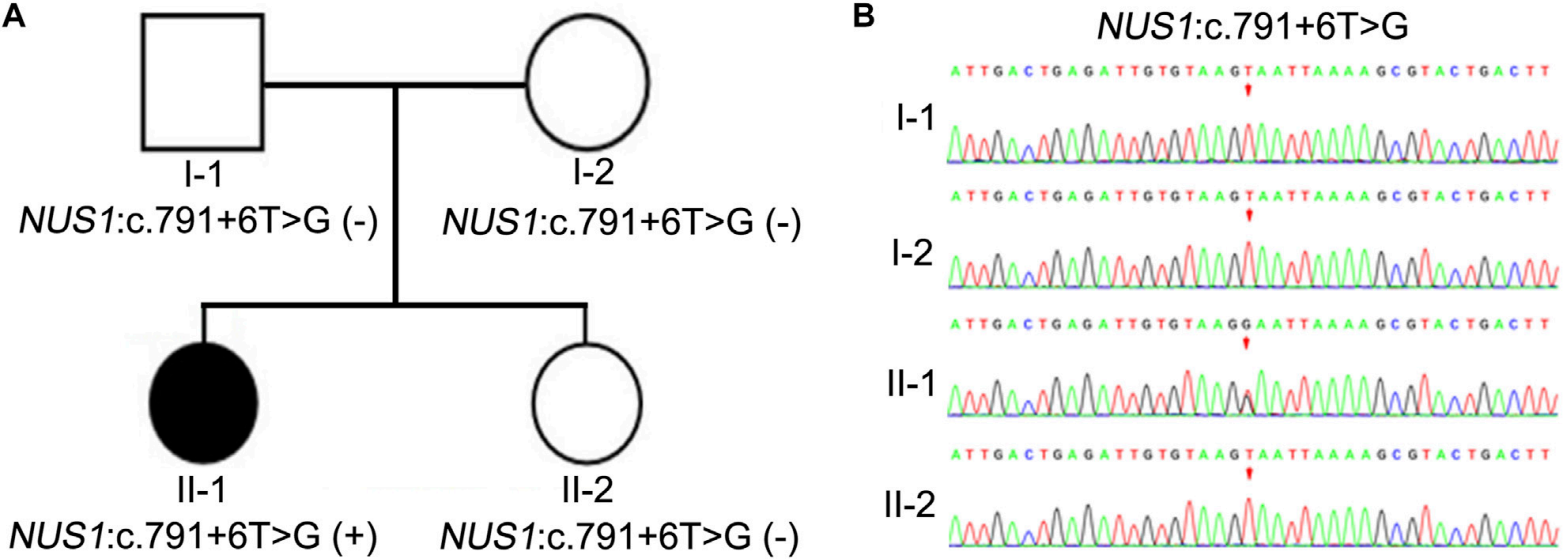
Pedigree and Sanger sequencing chromatograms of the identified pathogenic variant. **(A)** Schematic presentation of the familial pedigree of the patient. I-1, I-2, II-1, and II-2 represent the father, mother, proband, and sister, respectively. White fill represents an unaffected family member, while black fill represents an affected member. **(B)** Sanger sequencing chromatogram of *NUS1* in the family. The variant c. 791 + 6T>G in *NUS1* was identified in II-1.

In order to further investigate the potential genetic causes of the disorder, family whole-exome sequencing was performed on the proband and her parents. SNV and InDel results showed that there were two heterozygous variants of *NUS1*, namely, c.791 + 6T>G and c.537T>A. The missense variant c.537T>A was present in both the patient and her healthy father, but was absent in her sister and mother (Supplementary Figure S1), suggesting that it was not likely to be the cause of the disease. The other intronic variant, c.791 + 6T>G, was identified exclusively in the patient and was not found in the mother, father, or sister (Figure 2), supporting a co-segregation of the variant with disease. Additionally, this intronic variant was not found in the Shenzhou Genome database, the human Exon Database (ExAC), the reference population of the 1000 Genomes Project (1000G), or the Population Genome Mutation Frequency Database (gnomAD), suggesting that it was a novel variant. Therefore, we considered the variant c.791 + 6T>G to be the likely cause of the disease.

### Effect of the NUS1 c.791+6T>G variant on splicing

**FIGURE 3:**
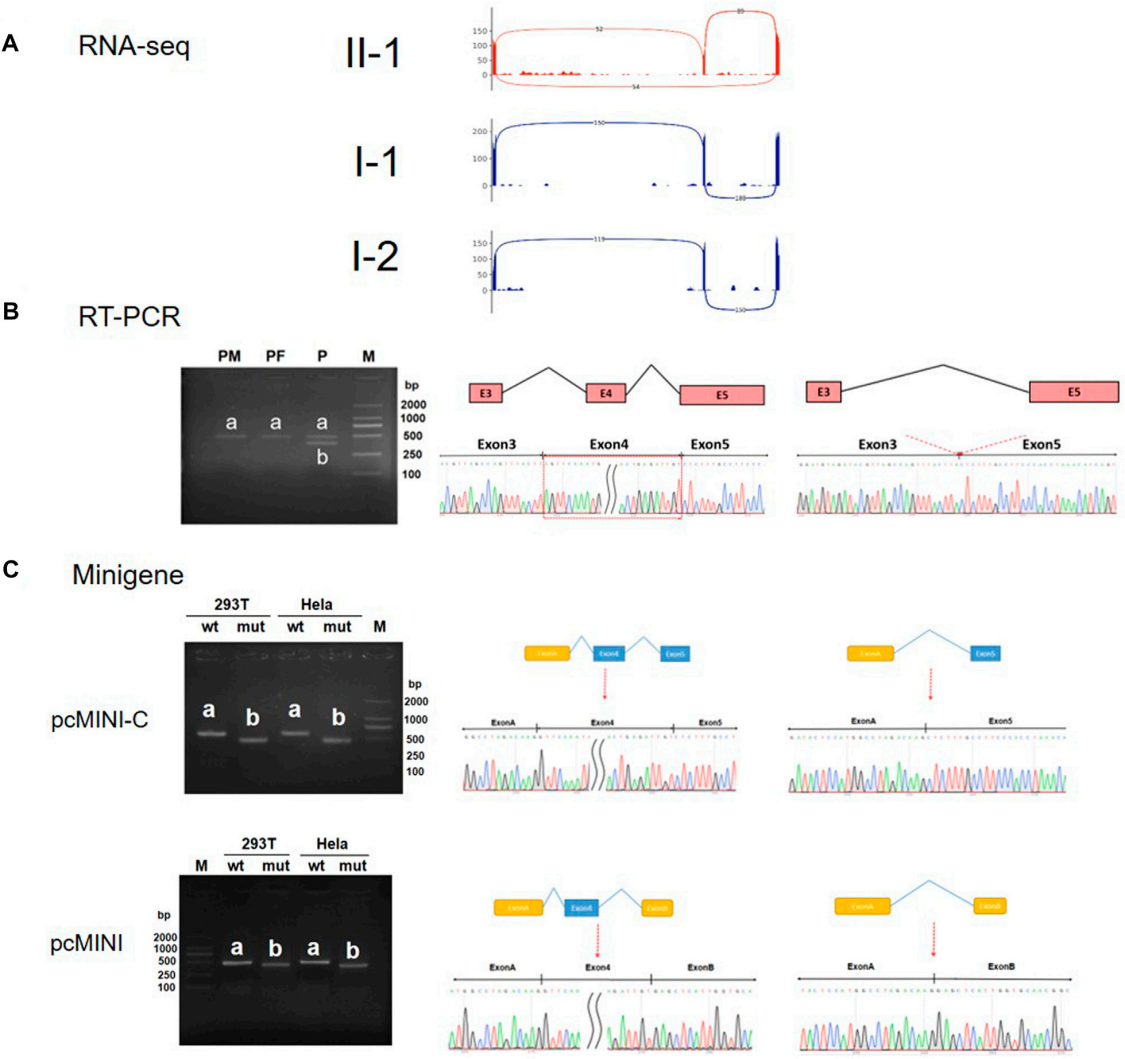
RNA-Seq, RT-PCR, and minigene assay analysis of the *NUS1* variant c. 791+6T>G. **(A)** Splicing events of the *NUS1* gene in the proband (red) and control (gray) identified via RNA-Seq. **(B)** Left: results of electrophoresis of the RT-PCR products of the family’s peripheral leukocytes; the bands amplified in samples from the parents (PM and PF) are labeled ‘a’ and the bands amplified in a sample from the proband (P) are labeled ‘b’. Right: the corresponding Sanger sequencing results of excised bands a and b from the RT-PCR assay. **(C)** The splicing products of WT and the c. 791 + 6T>G minigene constructs (pcMINI-C and pcMINI) are labeled ‘a’ and ‘b’. Left: RT-PCR electrophoresis results of the minigene assay; bands from the WT and c. 791 + 6T>G constructs are labeled ‘a’ and ‘b’, respectively, in both 293T and HeLa cells. Right: The results of Sanger sequencing correspond to the excised bands **(A)** and **(B)** in microarray gene analysis.

In order to verify the pathogenicity of NM_138,459.5: c.791 + 6T>G, *in vitro* and *in vivo* experiments were conducted to identify its functional consequences. Samples from the patient’s healthy parents were used as controls. RNA-Seq results showed that the patient (II-1) showed both inclusion and exclusion of Exon 4, whereas the healthy controls (I-1 and I-2) exhibited only normal inclusion of Exon 4 (Figure 3A). *In vivo* RT-PCR and Sanger sequencing confirmed that both the father and the mother had a single normal band ‘a’ (expected size 477bp), but the patient had two bands; band ‘a’ was the same size as expected, and band ‘b’ was smaller than band ‘a’ due to skipping of Exon 4 (Figure 3B). Then two *in vitro* expression constructs of Exon 4 (pcMINI-C and pcMINI) and the flanking sequences that contained the variant site were assembled and minigene splicing assays were carried out in 293T and HeLa cells. In the wild type constructs, there were bands consistent with the expected size for Exon 4 inclusion, while in the variant constructs, bands 100 bp smaller were observed due to skipping of Exon 4 (Figure 3C). Taken together, these experimental results suggest that the c.791 + 6T>G variant in *NUS1* does affect the normal splicing of mRNA, causing Exon 4 to be skipped.

### Interpretation of the variants based on the ACMG guidelines

The c.791 + 6T>G variant of *NUS1* gene is a new and rare splicing variant, and its detrimental impact on splicing has been demonstrated through *in vitro* and *in vivo* functional experiments. According to the pathogenicity classification guidelines of the American College of Medical Genetics and Genomics (ACMG), the c.791 + 6T>G variant is classified as a pathogenic variant. The pathogenicity rating (PS2+PM2+PS3 Moderate = P) was established as follows.

1) PS2 requires confirmation of the new variant through parental verification and absence of family history. The results of family exome sequencing confirmed that the c.791 + 6T>G variant is a *de novo* variant in this case.
2) PM2 requires absence of the variant in normal control populations, as documented in the ESP, 1000G, or EXAC databases (or its presence at very low frequency in the case of a recessive disease). The c.791 + 6T>G variant is not recorded in the aforementioned databases, meeting the requirement.
3) PS3 requires strong evidence from established functional studies, either *in vitro* or *in vivo*, supporting the detrimental effect on the gene or gene product. In this case, *in vitro* and *in vivo* functional tests have demonstrated that the c.791 + 6T>G variant of *NUS1* results in the skipping of Exon 4 and produces an mRNA product selectively shortened by 100 base pairs. This deletion causes a frameshift in the subsequent reading frame and introduces a premature termination codon (PTC) in Exon 5 (Supplementary Figure S2).

## Discussion

*NUS1* is located in 6q22.1 and encodes NgBR with a length of 293aa (Szafranski et al., 2015). *NUS1* is a relevant gene in multiple diseases, such as epilepsy and congenital glycosylation disorder, and its variants may cause a range of disease manifestations (Szafranski et al., 2015; Hamdan et al., 2017; Guo et al., 2018; Araki et al., 2020; Wirth et al., 2020; Courage et al., 2021; Fraiman et al., 2021; Gunzler and DeBrosse, 2021; Haginoya et al., 2021; Yu et al., 2021; Zhang et al., 2021; Monfrini et al., 2022; Ji et al., 2023). In this article, we have reported a non-canonical splicing site variant in the *NUS1* gene and further verified its functional effect on splicing.

Cis-prenyltransferase is a heterotetramer formed from two DHDDS-NgBR heterodimers, mutations to which have been identified as causing neurological diseases. NgBR is an auxiliary stationary subunit and DHDDS is a catalytic subunit of cis-prenyltransferase (Harrison et al., 2011; Guo et al., 2018). NgBR has no endogenous cis-PTase activity, but it can accelerate the activity of DHDDS 400-fold through direct interaction of its C-terminal with DHDDS active site residues and the substrates (Giladi et al., 2022). With the C-terminal truncated, the NgBR variant observed in this patient would not be able to interact with DHDDS or the substrates, thus drastically reducing the enzyme activity of cis-prenyltransferase (Courage et al., 2021). However, further studies with patient-derived fibroblast cells are needed to assess this functional defect in protein glycosylation.

We compared the symptoms of 17 other reported cases of *NUS1* mutation with the symptoms reported in our case study, and found that the phenotypes overlapped to varying degrees (Supplementary Table S1). More than half of the cases had symptoms similar to those reported in our case study, such as delayed movement, delayed speech, intellectual disability, cerebellar ataxia, tremors, and epileptic seizures, while fever-induced seizures and other symptoms were not common, occurring in only a small number of patients. It is worth noting that 83.33% of the cases were diagnosed with epilepsy-related diseases, indicating a relatively strong causal relationship between *NUS1* variants and epilepsy (Supplementary Figure S3; Supplementary Table S1).

The variant carried by the patient we have reported on here is a novel splice variant in *NUS1*. So far, six other *NUS1* splicing variants have been reported (Table 1). Among these, c.691+3dupA and c.691 + 1C>A both lead to 91 bp of Exon 3 being skipped and premature termination within Exon 4, but c.691+3dupA results in a larger reduction in mRNA level than c.691 + 1C>A. Previous publications in the literature have reported that nonsense-mediated mRNA decay (NMD) can degrade transcripts containing premature termination codons (PTCs). However, a small fraction of transcripts can escape NMD and remain undegraded, with potential variations in outcomes observed in different mutant forms and/or cell types (Yao et al., 2022; Chu et al., 2023; Munoz-Pujol et al., 2023; Zheng et al., 2023). Similar to c.691 + 1C>A, blood samples in our case of c.791 + 6T>G were collected to identify the consequent changes in mRNA. RNA-Seq and RT-PCR results indicated that mRNA level is not significantly reduced by c.791 + 6T>G (Figures 3A, B), resembling the case of c.691 + 1C>A. However, the patient in this case study exhibited less severe symptoms than those with c.691 + 1C>A variants, possibly because the c.791 + 6T>G variant in *NUS1* leads to deletion of Exon 4 at the C-terminal of NgBR, while the c.691 + 1C>A variant leads to deletion further upstream of NgBR. This difference in missing length may result in functional differences in NgBR.

**TABLE 1:** Information on *NUS1* splicing site variation.

Variant	Inheritance	Feature	Splicing results	PTC	NMD	Sampling
c.415 + 1G>A	*De novo*	Seizure	-	-	-	-
c.542-1G>A	*De novo*	CDG1AA	-	-	-	-
c.691 + 1C>A	*De novo*	Seizure	91bp deletion in Exon 3	Yes, in Exon 4	Not obvious	Blood
c.691+3dupA	*De novo*	Parkinson’s	91bp deletion in Exon 3	Yes, in Exon 4	Obvious	Fibroblast
c.692-1G>A	*De novo*	CDG1AA	-	-	-	-
c.692–2A>G	*De novo*	CDG1AA	-	-	-	-
c.791 + 6T>G^a^	*De novo*	Seizure	Exon 4 deletion	Yes, in Exon 5	Not obvious	Blood

^a^ The variant identified in this study.

To assess the functional consequence of our identified intronic variant, both *in vivo* (RNA-Seq/RT-PCR) and *in vitro* (minigene assay) analyses were performed. Patient blood sample is usually accessible for RNA-Seq/RT-PCR analysis of the whole transcriptome or certain genes of interest, which can be routine and quick to complete in diagnostic laboratories (Fraiman et al., 2021; Yao et al., 2022; Chu et al., 2023; Munoz-Pujol et al., 2023; Zheng et al., 2023). However, tissue-specific expression can limit this *in vivo* application, since many genes may not be expressed at a detectable level in blood cells (even though this is not the case for *NUS1*). Furthermore, a causal relationship between c.791 + 6T>G and the observed splicing change in *NUS1* cannot be formally established based only on *in vivo* results, because contributions from other genetic differences between the patient and her parents could not be ruled out. Therefore, minigene assay can help establish this causal connection by comparing the transcript products of two expression constructs that differ only at the variant site, although it takes time and effort to set up this assay. Due to a size limitation, minigene constructs only contain intronic regions and neighboring exons, which may not recapitulate all the necessary splicing components, so results from a minigene assay only serve as a proxy for *in vivo* splicing changes. Together, the consistent implications of the evidence from both *in vivo* and *in vitro* data in this case support a direct role of the identified variant c.791 + 6T>G in the disease outcome.

Currently, due to the small number of reported cases of *NUS1*-related disorders, in addition to the absence of detailed documentation and characterization for many of those cases, it is difficult to identify the underlying genotype–phenotype relationship. Moreover, there is no apparent pattern of drug efficacy across the cases with recorded drug treatment, where both ethnic and individual differences are present. We still need more detailed and accurate case reports, which will help in investigating the correlation between *NUS1* variants and disease prognosis, and will also lay a solid foundation for the development of accurate and rapid genetic diagnosis and treatment in the future.

## Data Availability

The original contributions presented in the study are included in the article/Supplementary Material, further inquiries can be directed to the corresponding authors.

## References

[B1] Araki K. Nakamura R. Ito D. Kato K. Iguchi Y. Sahashi K. (2020). NUS1 mutation in a family with epilepsy, cerebellar ataxia, and tremor. Epilepsy Res. 164, 106371. 10.1016/j.eplepsyres.2020.106371 32485575

[B3] Chu G. Li P. Zhao Q. He R. Zhao Y. (2023). Mutation spectrum of kallmann syndrome: Identification of five novel mutations across ANOS1 and FGFR1. Reprod. Biol. Endocrin 21, 23. 10.1186/s12958-023-01074-w PMC9976430 36859276

[B4] Courage C. Oliver K. L. Park E. J. Cameron J. M. Grabinska K. A. Muona M. (2021). Progressive myoclonus epilepsies-Residual unsolved cases have marked genetic heterogeneity including dolichol-dependent protein glycosylation pathway genes. Am. J. Hum. Genet. 108, 722–738. 10.1016/j.ajhg.2021.03.013 33798445 PMC8059372

[B5] Den K. Kudo Y. Kato M. Watanabe K. Doi H. Tanaka F. (2019). Recurrent NUS1 canonical splice donor site mutation in two unrelated individuals with epilepsy, myoclonus, ataxia and scoliosis - a case report. BMC Neurol. 19, 253. 10.1186/s12883-019-1489-x 31656175 PMC6815447

[B6] Fraiman P. Maia-de-Oliveira J. P. Moreira-Neto M. Godeiro-Junior C. (2021). Psychosis in NUS1 de novo mutation: New phenotypical presentation. Clin. Genet. 99, 475–476. 10.1111/cge.13867 33111323

[B7] Giladi M. Lisnyansky Bar-El M. Vankova P. Ferofontov A. Melvin E. Alkaderi S. (2022). Structural basis for long-chain isoprenoid synthesis by cis-prenyltransferases. Sci. Adv. 8, eabn1171. 10.1126/sciadv.abn1171 35584224 PMC9116609

[B8] Grabinska K. A. Edani B. H. Park E. J. Kraehling J. R. Sessa W. C. (2017). A conserved C-terminal RXG motif in the NgBR subunit of cis-prenyltransferase is critical for prenyltransferase activity. J. Biol. Chem. 292, 17351–17361. 10.1074/jbc.M117.806034 28842490 PMC5655512

[B9] Gunzler S. A. DeBrosse S. D. (2021). Generalized dystonia as a prominent feature in a case of NUS1 gene mutation. Can. J. Neurol. Sci. 48, 433–434. 10.1017/cjn.2020.204 32959737

[B10] Guo J. F. Zhang L. Li K. Mei J. P. Xue J. Chen J. (2018). Coding mutations in NUS1 contribute to Parkinson's disease. Proc. Natl. Acad. Sci. U.S.A. 115, 11567–11572. 10.1073/pnas.1809969115 30348779 PMC6233099

[B11] Haginoya K. Sekiguchi F. Munakata M. Yokoyama H. Hino-Fukuyo N. Uematsu M. (2021). A patient with a 6q22.1 deletion and a phenotype of non-progressive early-onset generalized epilepsy with tremor. Epilepsy Behav. Rep. 15, 100405. 10.1016/j.ebr.2020.100405 33437959 PMC7786037

[B12] Hamdan F. F. Myers C. T. Cossette P. Lemay P. Spiegelman D. Laporte A. D. (2017). High rate of recurrent de novo mutations in developmental and epileptic encephalopathies. Am. J. Hum. Genet. 101, 664–685. 10.1016/j.ajhg.2017.09.008 29100083 PMC5673604

[B13] Harrison K. D. Park E. J. Gao N. Kuo A. Rush J. S. Waechter C. J. (2011). Nogo-B receptor is necessary for cellular dolichol biosynthesis and protein N-glycosylation. EMBO J. 30, 2490–2500. 10.1038/emboj.2011.147 21572394 PMC3116281

[B14] Jaeken J. Peanne R. (2017). What is new in CDG? J. Inherit. Metab. Dis. 40, 569–586. 10.1007/s10545-017-0050-6 28484880

[B15] Ji C. Zhao J. Zhang J. Wang K. (2023). Novel NUS1 variant in a Chinese patient with progressive myoclonus epilepsy: A case report and systematic review. Neurol. Sci. 10.1007/s10072-023-06851-4 37249665

[B16] Monfrini E. Miller C. Frucht S. J. Di Fonzo A. Riboldi G. M. (2022). Progressive myoclonus without epilepsy due to a NUS1 frameshift insertion: Dyssynergia cerebellaris myoclonica revisited. Park. Relat. D. 98, 53–55. 10.1016/j.parkreldis.2022.03.016 35472621

[B17] Munoz-Pujol G. Ortigoza-Escobar J. D. Paredes-Fuentes A. J. Jou C. Ugarteburu O. Gort L. (2023). Leigh syndrome is the main clinical characteristic of PTCD3 deficiency. Brain Pathol. 33, e13134. 10.1111/bpa.13134 36450274 PMC10154364

[B18] Park E. J. Grabinska K. A. Guan Z. Stranecky V. Hartmannova H. Hodanova K. (2014). Mutation of Nogo-B receptor, a subunit of cis-prenyltransferase, causes a congenital disorder of glycosylation. Cell Metab. 20, 448–457. 10.1016/j.cmet.2014.06.016 25066056 PMC4161961

[B19] Riboldi G. M. Monfrini E. Stahl C. Frucht S. J. (2022). NUS1 and epilepsy-myoclonus-ataxia syndrome: An under-recognized entity? Tremor Other Hyperkinet Mov. (N Y) 12, 21. 10.5334/tohm.696 35949226 PMC9205445

[B20] Szafranski P. Von Allmen G. K. Graham B. H. Wilfong A. A. Kang S. H. Ferreira J. A. 6q22.1 microdeletion and susceptibility to pediatric epilepsy. Eur. J. Hum. Genet. (2015) 23: 173–179. 10.1038/ejhg.2014.75 24824130 PMC4297903

[B21] Wirth T. Tranchant C. Drouot N. Keren B. Mignot C. Cif L. (2020). Increased diagnostic yield in complex dystonia through exome sequencing. Park. Relat. D. 74, 50–56. 10.1016/j.parkreldis.2020.04.003 32334381

[B22] Yao Y. Deng S. Zhu F. (2022). Prenatal detection of novel compound heterozygous splice site variants of the KIAA0825 gene in a fetus with postaxial polydactyly type A. Genes 13, 1230. 10.3390/genes13071230 35886013 PMC9316509

[B23] Yu S. H. Wang T. Wiggins K. Louie R. J. Merino E. F. Skinner C. (2021). Lysosomal cholesterol accumulation contributes to the movement phenotypes associated with NUS1 haploinsufficiency. Genet. Med. 23, 1305–1314. 10.1038/s41436-021-01137-6 33731878 PMC8263489

[B24] Zhang P. Cui D. Liao P. Yuan X. Yang N. Zhen Y. (2021). Case Report: Clinical features of a Chinese boy with epileptic seizures and intellectual disabilities who carries a truncated NUS1 variant. Front. Pediatr. 9, 725231. 10.3389/fped.2021.725231 34532305 PMC8438189

[B25] Zheng F. Lin Z. Hu Y. Shi X. Zhao Q. Lin Z. (2023). Identification of a novel non-canonical splice-site variant in ABCD1. J. Clin. Med. 12, 473. 10.3390/jcm12020473 36675402 PMC9863105

