# Supplementary material for: Case report: splicing effect of a novel heterozygous variant of the NUS1 gene in a child with epilepsy
Source: Front Genet. 2023 Jul 4;14:1224949. doi: 10.3389/fgene.2023.1224949 (PMC10352580; doi:10.3389/fgene.2023.1224949)
Supplement: Supplementary file 1 [file DataSheet1.docx]

**Table S1 A comparative analysis of 17 reported cases of *NUS1* mutation and the cases in this study**

| NUS1 variant | c.692-1 G>A | p.Thr261Serfs*9 | c.302T>A | c.305G>A | c.22-23insA | c. 104G>A | c.734G＞T | c.752T＞G | c.415+1G＞A | c.743delA | c.128-141dup | exon2 deletion | p.R290C | P.R290H | c.691+1C＞A | c.691+1C＞A | c.691+3dupA | 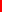c.791+6T＞G | Percentage |
| --- | --- | --- | --- | --- | --- | --- | --- | --- | --- | --- | --- | --- | --- | --- | --- | --- | --- | --- | --- |
| PMID | 33111323 | 35472621 | 37249665 | 32334381 | 32485575 | 32959737 | 33731878 | 33731878 | 33731878 | 29100083 | 29100083 | 29100083 | 35949226 | 31656175 | 31656175 | 31656175 | 31656175 | this study |  |
| jerks | yes | yes | / | / | / | / | / | / | / | yes | / | / | / | / | / | / | / | / | 16.67% |
| convulsion | / | / | / | / | / | / | / | / | / | / | / | / | / | / | yes | / | / | / | 5.56% |
| ferbile | / | / | / | / | / | / | / | / | yes | / | / | / | / | / | yes | / | / | / | 11.11% |
| hypotonia | / | / | / | / | / | / | / | / | / | / | / | / | yes | yes | / | / | / | / | 11.11% |
| dysmorphic features | / | / | / | / | / | / | / | / | / | / | / | / | yes | yes | / | / | / | / | 11.11% |
| tremors | / | / | / | yes | yes | yes | / | / | / | / | / | / | yes | / | / | / | / | yes | 27.78% |
| scoliosis | / | / | / | / | / | / | / | / | / | / | / | / | yes | yes | yes | yes | / | / | 22.22% |
| clonic | / | / | / | / | / | / | yes | / | yes | / | yes | yes | / | / | yes | / | / | / | 27.78% |
| ataxia | yes | / | / | / | / | yes | yes | yes | / | yes | / | / | / | / | yes | yes | yes | / | 44.44% |
| motor delay | / | / | / | / | / | / | / | / | yes | yes | yes | yes | yes | / | yes | / | / | yes | 44.44% |
| speech delay | / | / | / | / | / | yes | yes | yes | yes | yes | yes | / | / | / | yes | yes | / | yes | 50.00% |
| intellectual disability | yes | yes | yes | yes | yes | yes | yes | yes | / | yes | yes | yes | yes | / | yes | yes | / | / | 77.78% |
| myoclonic | yes | yes | yes | yes | yes | yes | / | yes | yes | yes | yes | yes | yes | / | yes | yes | / | yes | 83.33% |
| seizures | yes | / | yes | / | yes | yes | yes | yes | yes | yes | yes | yes | yes | yes | yes | yes | / | yes | 83.33% |

Red represents the variation found in this study

**
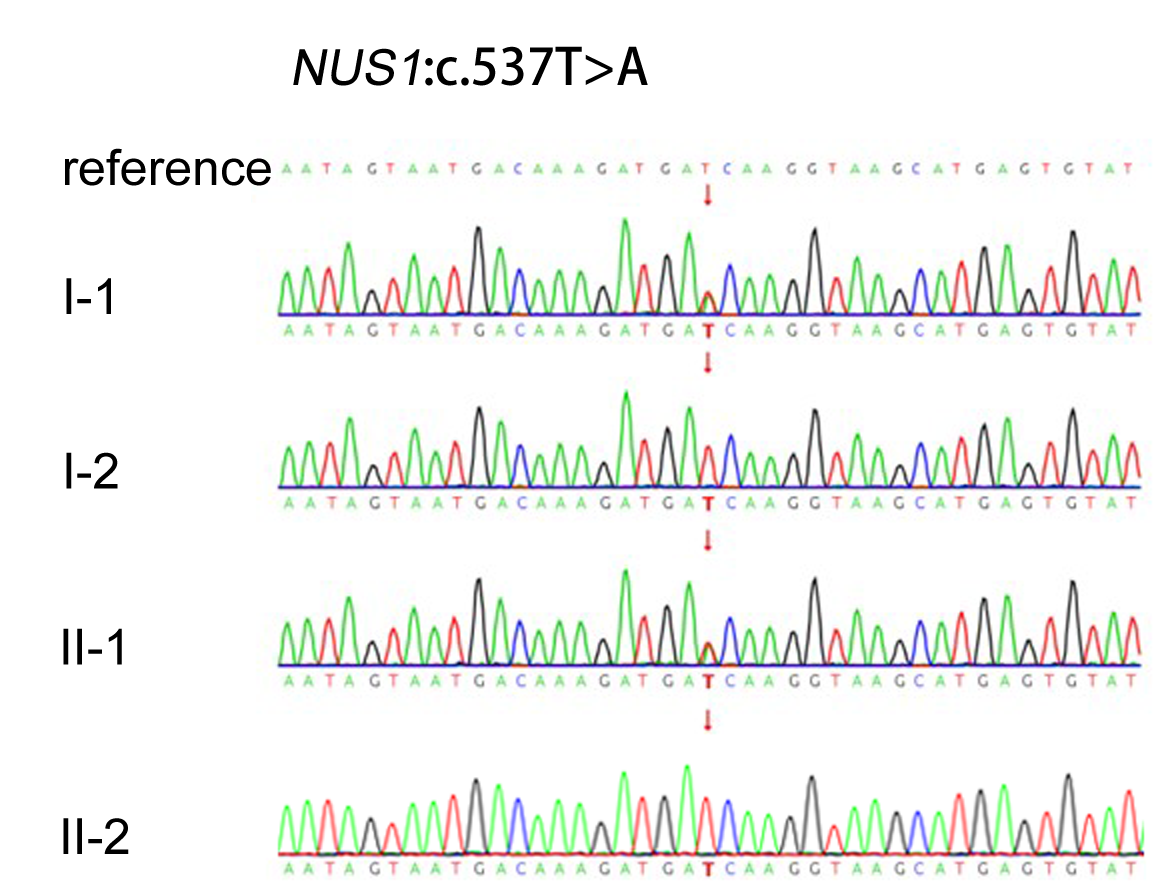
**

**Figure S1 Sanger sequencing chromatogram of the *NUS1* variant in the family.** The variant c.537T>A in *NUS1* is identified in II-1 and I-1.


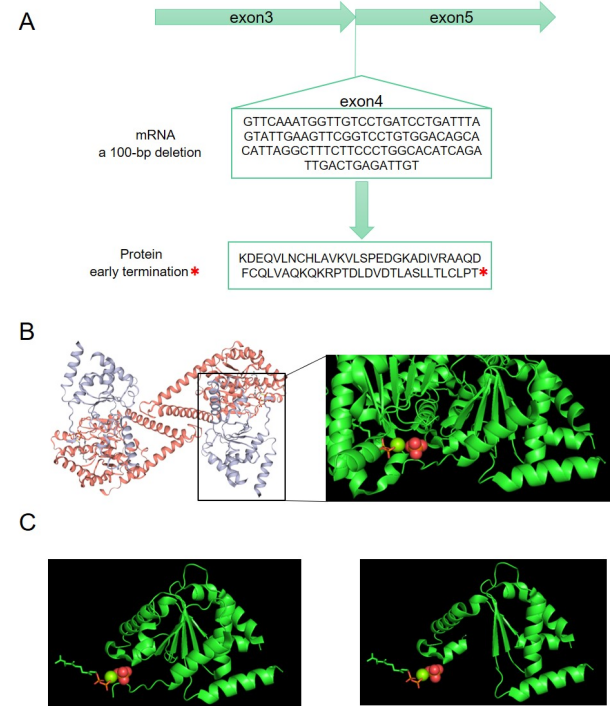


**Figure S2. Prediction of the 3D structure of the protein with *NUS1* splicing mutation.** (A) RNA-seq reveals a 100-base pair deletion between exon 3 and exon 5. The deletion of exon 4 results in a change in the reading frame and premature termination of exon 5. (B) Structural diagram of the dehydrodipicolinate synthase complex (left); the magnified view (right) shows the interaction between the C-terminus of NgBR and the active site, where the green spheres represent magnesium ions, and the red spheres and orange stick represent phosphate ions and the small molecule FPP, respectively. (C) Schematic representation of the interaction between normal NgBR and small molecules (left) and truncated NgBR and small molecules (right).


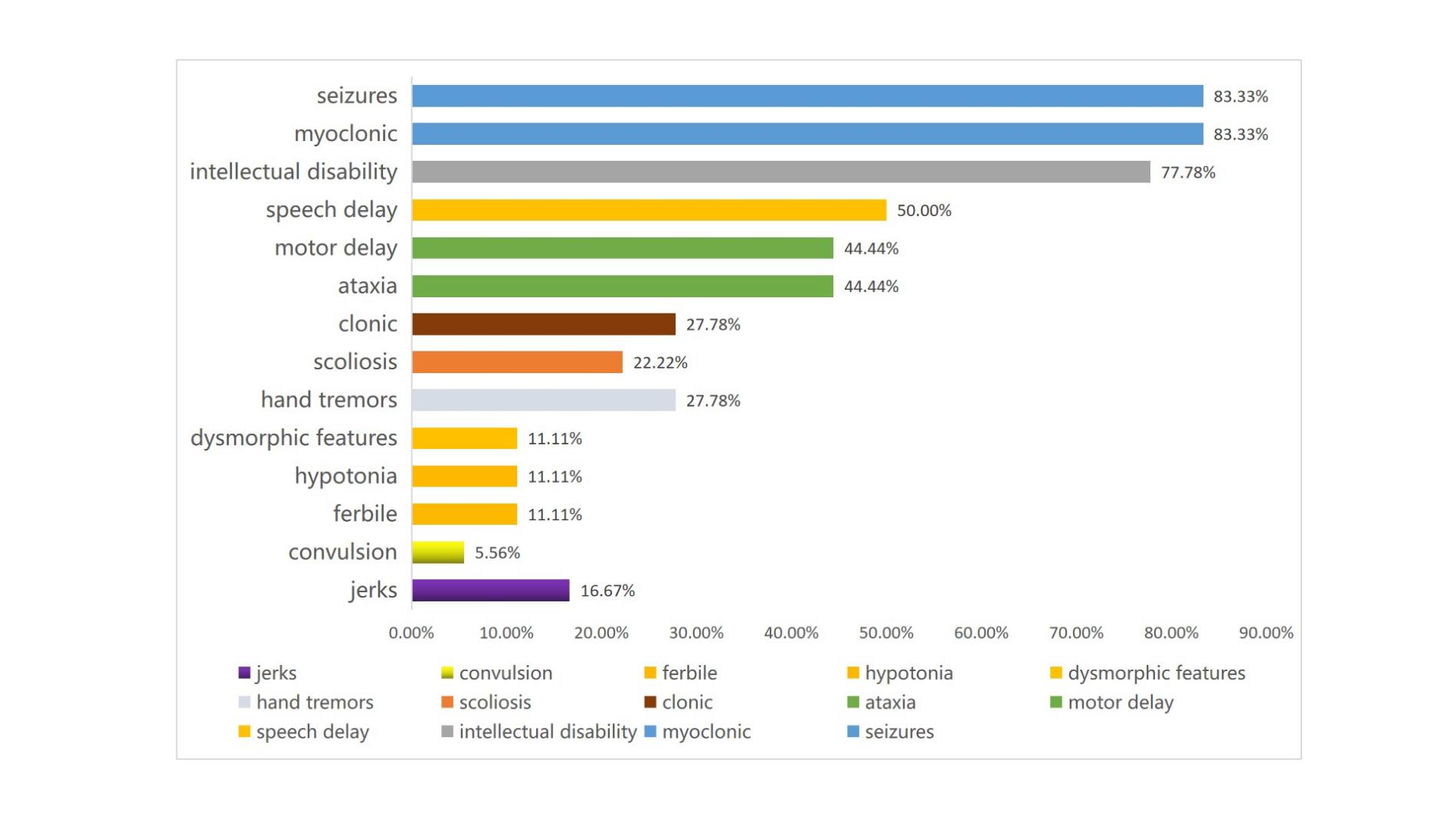


**Figure S3 Symptom phenotype statistics of 18 epileptic patients**
